# “Irregularities” in the Quarantine Department

**Published:** 1902-02

**Authors:** 


### TEXAS MEDICAL JOURNAL.

AUSTIN, TEXAS.

A MONTHLY JOURNAL OF MEDICINE AND SURGERY.

EDITED AND PUBLISHED BY
F. E. DANIEL, M. D.

ASSOCIATE EDITOR:
WITTEN BOOTH RUSS, M. D.,
San Antonio, Texas.

Published Monthly at Austin, Texas. Subscription price $1.00 a year in advance.

Eastern Representative: John Guy Monihan, St. Paul Building, 220 Broadway, New York City.

Official organ of the State Association of Health Officers, the West Texas Medical Association, the Houston District Medical Association, the Austin District Medical Society, the Brazos Valley Medical Association, the Galveston County Medical Society, and several others.

### "IRREGULARITIES" IN THE QUARANTINE DEPARTMENT.

In the last issue of the JOURNAL, I gave some of the facts in relation to the defalcation or misappropriation of funds belonging to the Quarantine Department, substantially as they were related to me by State Health Officer Tabor, and gathered from a brief conversation with the gentleman who was at that time examining the books, and also as stated by Dr. Tabor in a communication to the Austin daily press. At that time the investigation had not revealed all the facts, nor the methods used by ex-Secretary Jones, nor was the extent of the shortage known. It is learned now that it will exceed $7000. The report of the expert accountant who examined the books is in the hands of the Governor, and has not yet been made public. Nor is it known whether or not either Dr. Blunt or his bondsmen are responsible pecuniarly for the shortage. Dr. Blunt has written me a letter under date of January 15th, complaining that I did him an injustice in the article referred to (wherein, I cannot see), and I take this occasion to disclaim any intention of doing so, and I regret that he has so construed it. I publish with pleasure his letter:

LOCKHART, TEXAS, Jan. 15th, 1902.

*Dr. F. E. Daniel, Austin, Texas.*

DEAR DOCTOR: I write you in reference to the article headed "Irregularities in the Texas Quarantine Department," appearing in your January issue of the "Red Back." I do not believe you would knowingly misrepresent the facts connected with Dr. Jones' defalcation of State funds. Therefore, I make the following statement:

As you well know, I had no money under my control except the fumigating fund, and that fund is not short one cent, as the defalcations that were made were not on or from that fund, but were made by forgeries, and the money collected from the Comptroller's Department. You will please call on Mr. Sam McCullough, who, as you know, was appointed by the Governor expert accountant to look into Jones' forgeries, and he will inform you that the above are the facts.

As you stated in your article, Dr. Jones had complete charge of my books and accounts, but he *did not* have authority, either verbally or written, to sign vouchers in my department. On the other hand, Governor Sayers required that I should first sign all vouchers myself and submit them to him for his signature, and when money had to be drawn out of the fumigating fund the check was always signed first by W. F. Blunt, State Health Officer, and then J. D. Sayers, Governor, as the money was deposited in bank in the names of W. F. Blunt and J. D. Sayers, and could not be drawn without both of our signatures to the check.

Dr. Tackaberry's defalcations at Sabine Pass occurred this way: He carried a hand three months on his pay roll at $60 a month which he did not have employed, but swore to this pay roll, and collected the amount by swearing to a false pay roll. He also acknowledged to having collected $10 from a ship that he never reported to Quarantine Department, so you readily see that this fraud does not reflect on me in the least manner. He was an appointee of the Governor.

I would be pleased, and think it due me, if you will look into this matter and make a statement in your February JOURNAL that will do me full justice. If you will hand this letter to Mr. McCullough and request him for me to give you the facts, I know he will willingly do so.

W. F. BLUNT, M. D.
Per F.

[Mr. McCullough, at this writing, is not at liberty to make a statement for publication, as his report to the Governor has not yet been published.—ED.]

GOOD NEWS.—The time and place of meeting of the Texas State Medical Association have been changed from El Paso, April 22-25, to Dallas, May 6, 7, 8 and 9.

When the physicians of El Paso, who had invited the Association to meet in their city (and their invitation was accepted without due consideration) were convinced that it would be detrimental to the best interests of the Association to hold its meeting so far away from the center of medical population, with courtesy most commendable consented to yield the point contended for by the "Red Back," and left the selection of a place of meeting to the members of the Association. Dr. Taylor Hudson, through Secretary West, sent an addressed postal card to each member, with a request to indicate his preference as to place of meeting, and President Hudson informs us that "Dallas having been the expressed choice of the majority," the meeting will be held in that city, but owing to conflict with the meeting of the ex-Confederate Surgeons," and the reunion of the C. V. Association, which meetings are to take place in Dallas April 22, 23, 24 and 25, the date of the meeting of the State Medical Association has been changed to May 6, 7, 8 and 9.

The "Red Back" extends congratulations to its constituents, especially its country contingent, living so remote from El Paso that the meeting, if it had been held there, would have been out of their reach, and shakes hands with itself in congratulation, for, but for its early and persistent efforts to bring about the change, it would not have been made. Thus, the JOURNAL has had another opportunity to demonstrate its devotion to the interests and welfare of our organization, as well as an eye to that of its readers, the rank and file, the bone and sinew of the Association; the "boys from the forks of the creek." I trust and believe that those in whose interests this change has been advocated and brought about will show their appreciation by attending the meeting. It was a critical period in the life of the Association, and it has now been safely passed. The Constitution is to be changed, and a full and free discussion and a representative vote on the subject are desired. Let there be a "gathering of the clans,"—a full and representative meeting, and a rousing good time,—when the day's work is done. Dallas knows how to handle a crowd, and how to entertain, and every member of the Association who has ever attended a meeting there recollects the occasion,—and remembers the delightful hospitality of the people and the profession. The meeting will take place in the early days of glorious May,—when Spring has just donned her lovely attire and is splurging in her new Easter bonnet. With a smile and welcome she will help the doctors and their wives to "receive." Let every fellow, therefore, put on his best, and with purse and script and no thought for the morrow, turn out —turn out; take a rest, and give his horse—and his patients—a rest!

